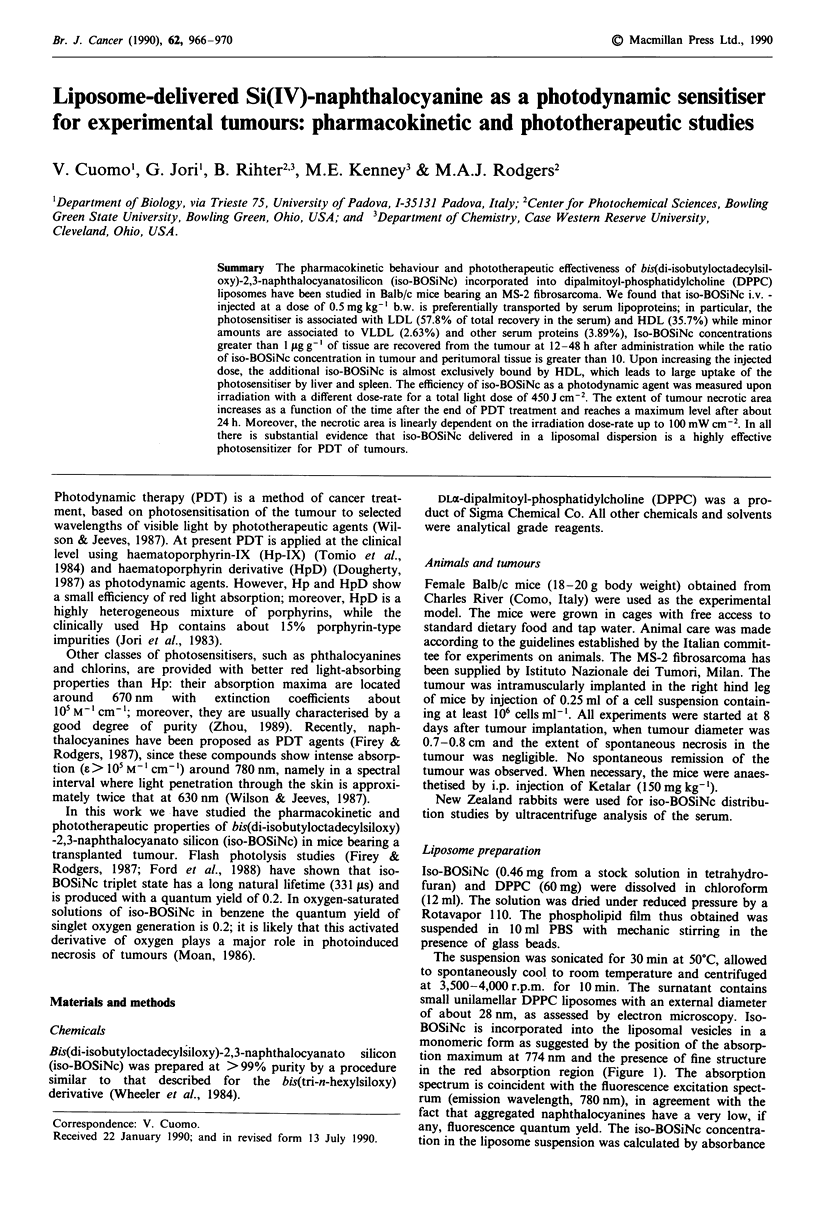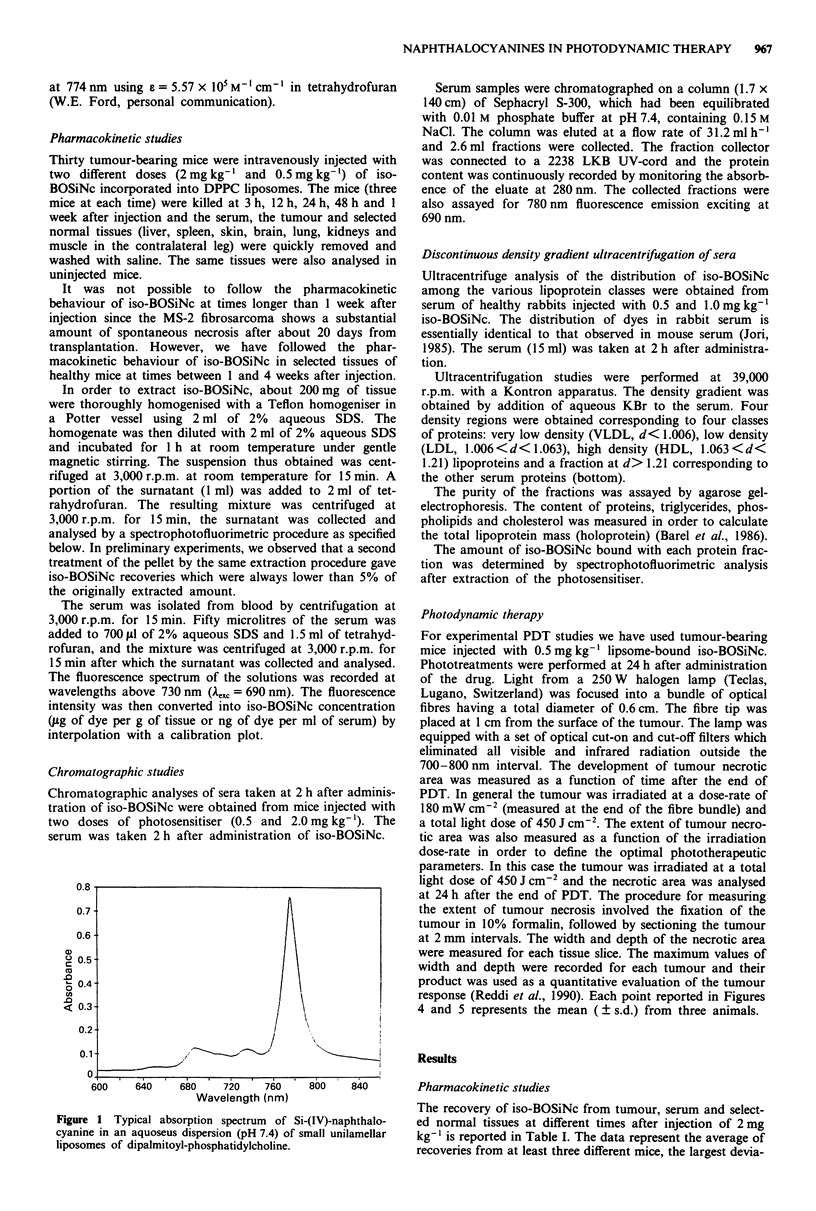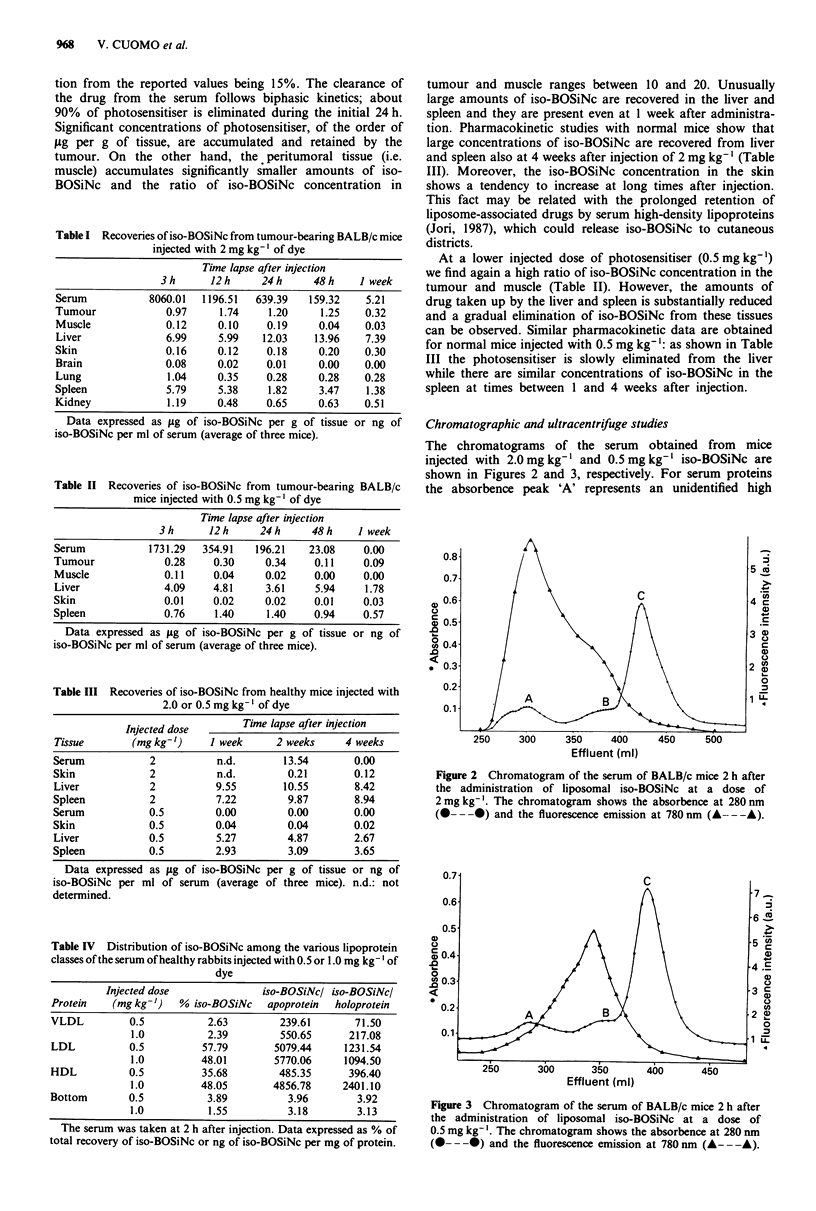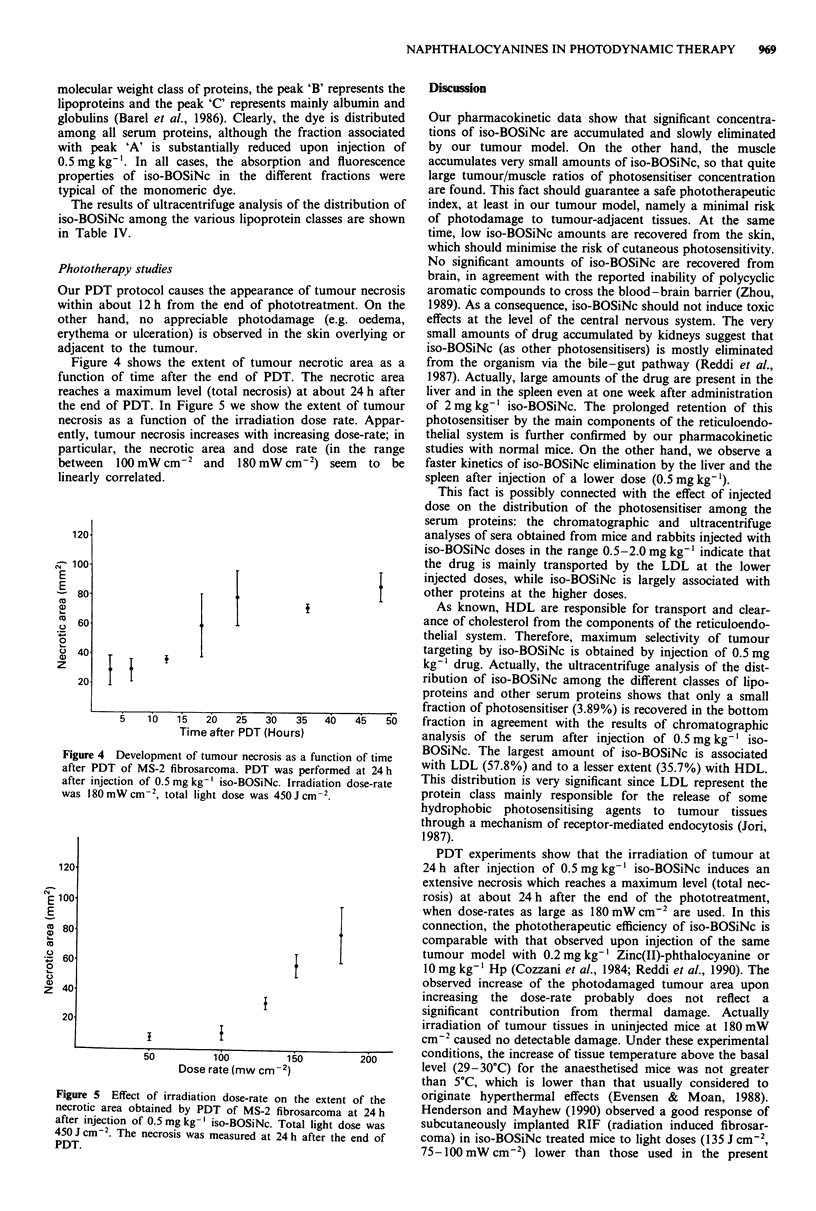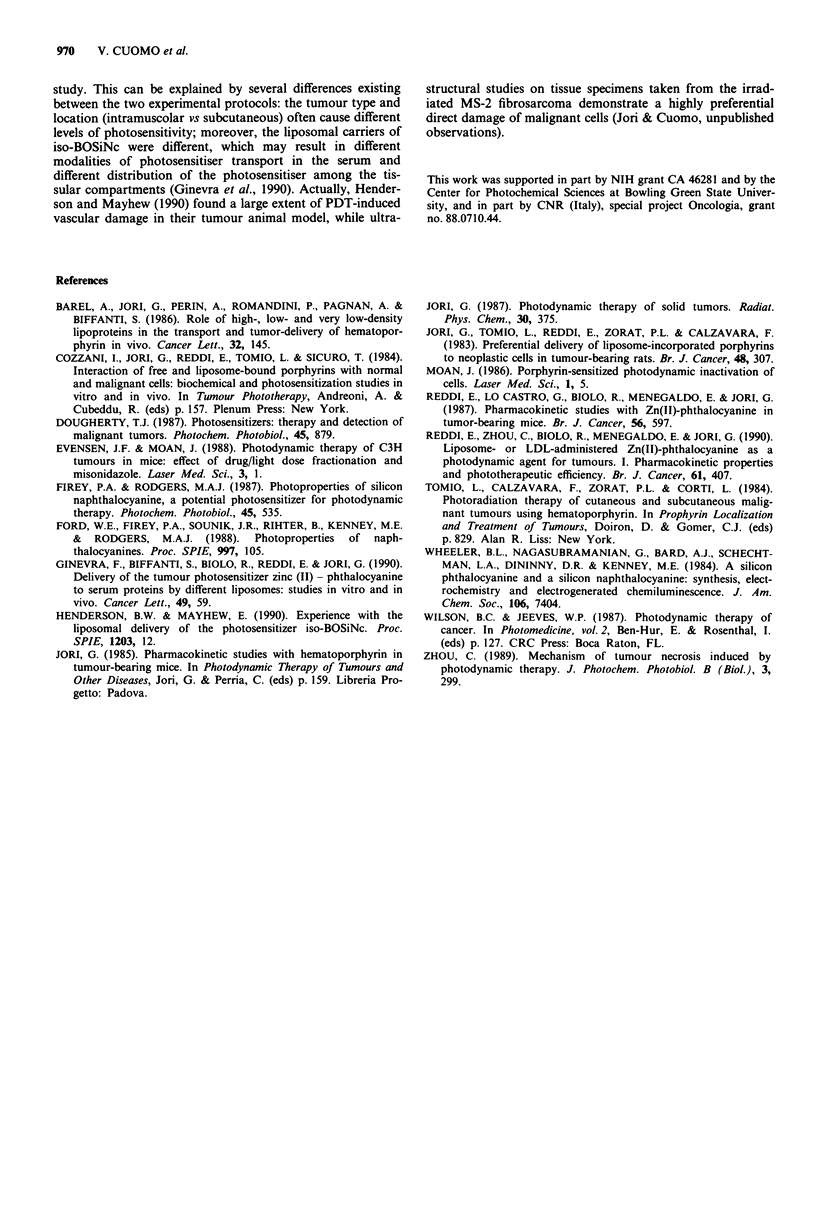# Liposome-delivered Si(IV)-naphthalocyanine as a photodynamic sensitiser for experimental tumours: pharmacokinetic and phototherapeutic studies.

**DOI:** 10.1038/bjc.1990.418

**Published:** 1990-12

**Authors:** V. Cuomo, G. Jori, B. Rihter, M. E. Kenney, M. A. Rodgers

**Affiliations:** Department of Biology, University of Padova, Italy.

## Abstract

The pharmacokinetic behaviour and phototherapeutic effectiveness of bis(di-isobutyloctadecylsil-oxy)-2,3-naphthalocyanatosilicon (iso-BOSiNc) incorporated into dipalmitoyl-phosphatidylcholine (DPPC) liposomes have been studied in Balb/c mice bearing an MS-2 fibrosarcoma. We found that iso-BOSiNc i.v.-injected at a dose of 0.5 mg kg-1 b.w. is preferentially transported by serum lipoproteins; in particular, the photosensitiser is associated with LDL (57.8% of total recovery in the serum) and HDL (35.7%) while minor amounts are associated to VLDL (2.63%) and other serum proteins (3.89%), Iso-BOSiNc concentrations greater than 1 microgram g-1 of tissue are recovered from the tumour at 12-48 h after administration while the ratio of iso-BOSiNc concentration in tumour and peritumoral tissue is greater than 10. Upon increasing the injected dose, the additional iso-BOSiNc is almost exclusively bound by HDL, which leads to large uptake of the photosensitiser by liver and spleen. The efficiency of iso-BOSiNc as a photodynamic agent was measured upon irradiation with a different dose-rate for a total light dose of 450 J cm-2. The extent of tumour necrotic area increases as a function of the time after the end of PDT treatment and reaches a maximum level after about 24 h. Moreover, the necrotic area is linearly dependent on the irradiation dose-rate up to 100 mW cm-2. In all there is substantial evidence that iso-BOSiNc delivered in a liposomal dispersion is a highly effective photosensitizer for PDT of tumours.


Br. J. Cancer (1990), 62, 966-970 © Macmillan Press Ltd., 1990

### Liposome-delivered Si(IV)-naphthalocyanine as a photodynamic sensitiser for experimental tumours: pharmacokinetic and phototherapeutic studies

V. Cuomo[1], G. Jori[1], B. Rihter[2,3], M.E. Kenney[3] & M.A.J. Rodgers[2]

[1]*Department of Biology, via Trieste 75, University of Padova, I-35131 Padova, Italy;* [2]*Center for Photochemical Sciences, Bowling Green State University, Bowling Green, Ohio, USA; and* [3]*Department of Chemistry, Case Western Reserve University, Cleveland, Ohio, USA.*

**Summary** The pharmacokinetic behaviour and phototherapeutic effectiveness of *bis*(di-isobutyloctadecylsiloxy)-2,3-naphthalocyanatosilicon (iso-BOSiNc) incorporated into dipalmitoyl-phosphatidylcholine (DPPC) liposomes have been studied in Balb/c mice bearing an MS-2 fibrosarcoma. We found that iso-BOSiNc i.v. - injected at a dose of 0.5 mg $kg^{-1}$ b.w. is preferentially transported by serum lipoproteins; in particular, the photosensitiser is associated with LDL (57.8% of total recovery in the serum) and HDL (35.7%) while minor amounts are associated to VLDL (2.63%) and other serum proteins (3.89%), Iso-BOSiNc concentrations greater than 1 $\mu g\ g^{-1}$ of tissue are recovered from the tumour at 12–48 h after administration while the ratio of iso-BOSiNc concentration in tumour and peritumoral tissue is greater than 10. Upon increasing the injected dose, the additional iso-BOSiNc is almost exclusively bound by HDL, which leads to large uptake of the photosensitiser by liver and spleen. The efficiency of iso-BOSiNc as a photodynamic agent was measured upon irradiation with a different dose-rate for a total light dose of 450 J $cm^{-2}$. The extent of tumour necrotic area increases as a function of the time after the end of PDT treatment and reaches a maximum level after about 24 h. Moreover, the necrotic area is linearly dependent on the irradiation dose-rate up to 100 mW $cm^{-2}$. In all there is substantial evidence that iso-BOSiNc delivered in a liposomal dispersion is a highly effective photosensitizer for PDT of tumours.

Photodynamic therapy (PDT) is a method of cancer treatment, based on photosensitisation of the tumour to selected wavelengths of visible light by phototherapeutic agents (Wilson & Jeeves, 1987). At present PDT is applied at the clinical level using haematoporphyrin-IX (Hp-IX) (Tomio *et al.*, 1984) and haematoporphyrin derivative (HpD) (Dougherty, 1987) as photodynamic agents. However, Hp and HpD show a small efficiency of red light absorption; moreover, HpD is a highly heterogeneous mixture of porphyrins, while the clinically used Hp contains about 15% porphyrin-type impurities (Jori *et al.*, 1983).

Other classes of photosensitisers, such as phthalocyanines and chlorins, are provided with better red light-absorbing properties than Hp: their absorption maxima are located around 670 nm with extinction coefficients about $10^5\ M^{-1}\ cm^{-1}$; moreover, they are usually characterised by a good degree of purity (Zhou, 1989). Recently, naphthalocyanines have been proposed as PDT agents (Firey & Rodgers, 1987), since these compounds show intense absorption ($\varepsilon > 10^5\ M^{-1}\ cm^{-1}$) around 780 nm, namely in a spectral interval where light penetration through the skin is approximately twice that at 630 nm (Wilson & Jeeves, 1987).

In this work we have studied the pharmacokinetic and phototherapeutic properties of *bis*(di-isobutyloctadecylsiloxy) -2,3-naphthalocyanato silicon (iso-BOSiNc) in mice bearing a transplanted tumour. Flash photolysis studies (Firey & Rodgers, 1987; Ford *et al.*, 1988) have shown that iso-BOSiNc triplet state has a long natural lifetime (331 μs) and is produced with a quantum yield of 0.2. In oxygen-saturated solutions of iso-BOSiNc in benzene the quantum yield of singlet oxygen generation is 0.2; it is likely that this activated derivative of oxygen plays a major role in photoinduced necrosis of tumours (Moan, 1986).

### Materials and methods

### *Chemicals*

*Bis*(di-isobutyloctadecylsiloxy)-2,3-naphthalocyanato silicon (iso-BOSiNc) was prepared at >99% purity by a procedure similar to that described for the *bis*(tri-*n*-hexylsiloxy) derivative (Wheeler *et al.*, 1984).

Correspondence: V. Cuomo.
Received 22 January 1990; and in revised form 13 July 1990.

DLα-dipalmitoyl-phosphatidylcholine (DPPC) was a product of Sigma Chemical Co. All other chemicals and solvents were analytical grade reagents.

### *Animals and tumours*

Female Balb/c mice (18–20 g body weight) obtained from Charles River (Como, Italy) were used as the experimental model. The mice were grown in cages with free access to standard dietary food and tap water. Animal care was made according to the guidelines established by the Italian committee for experiments on animals. The MS-2 fibrosarcoma has been supplied by Istituto Nazionale dei Tumori, Milan. The tumour was intramuscularly implanted in the right hind leg of mice by injection of 0.25 ml of a cell suspension containing at least $10^6$ cells $ml^{-1}$. All experiments were started at 8 days after tumour implantation, when tumour diameter was 0.7–0.8 cm and the extent of spontaneous necrosis in the tumour was negligible. No spontaneous remission of the tumour was observed. When necessary, the mice were anaesthetised by i.p. injection of Ketalar (150 mg $kg^{-1}$).

New Zealand rabbits were used for iso-BOSiNc distribution studies by ultracentrifuge analysis of the serum.

### *Liposome preparation*

Iso-BOSiNc (0.46 mg from a stock solution in tetrahydrofuran) and DPPC (60 mg) were dissolved in chloroform (12 ml). The solution was dried under reduced pressure by a Rotavapor 110. The phospholipid film thus obtained was suspended in 10 ml PBS with mechanic stirring in the presence of glass beads.

The suspension was sonicated for 30 min at 50°C, allowed to spontaneously cool to room temperature and centrifuged at 3,500–4,000 r.p.m. for 10 min. The surnatant contains small unilamellar DPPC liposomes with an external diameter of about 28 nm, as assessed by electron microscopy. Iso-BOSiNc is incorporated into the liposomal vesicles in a monomeric form as suggested by the position of the absorption maximum at 774 nm and the presence of fine structure in the red absorption region (Figure 1). The absorption spectrum is coincident with the fluorescence excitation spectrum (emission wavelength, 780 nm), in agreement with the fact that aggregated naphthalocyanines have a very low, if any, fluorescence quantum yeld. The iso-BOSiNc concentration in the liposome suspension was calculated by absorbance at 774 nm using $\varepsilon = 5.57 \times 10^5$ $M^{-1}$ $cm^{-1}$ in tetrahydrofuran (W.E. Ford, personal communication).

*Pharmacokinetic studies*

Thirty tumour-bearing mice were intravenously injected with two different doses (2 mg $kg^{-1}$ and 0.5 mg $kg^{-1}$) of iso-BOSiNc incorporated into DPPC liposomes. The mice (three mice at each time) were killed at 3 h, 12 h, 24 h, 48 h and 1 week after injection and the serum, the tumour and selected normal tissues (liver, spleen, skin, brain, lung, kidneys and muscle in the contralateral leg) were quickly removed and washed with saline. The same tissues were also analysed in uninjected mice.

It was not possible to follow the pharmacokinetic behaviour of iso-BOSiNc at times longer than 1 week after injection since the MS-2 fibrosarcoma shows a substantial amount of spontaneous necrosis after about 20 days from transplantation. However, we have followed the pharmacokinetic behaviour of iso-BOSiNc in selected tissues of healthy mice at times between 1 and 4 weeks after injection.

In order to extract iso-BOSiNc, about 200 mg of tissue were thoroughly homogenised with a Teflon homogeniser in a Potter vessel using 2 ml of 2% aqueous SDS. The homogenate was then diluted with 2 ml of 2% aqueous SDS and incubated for 1 h at room temperature under gentle magnetic stirring. The suspension thus obtained was centrifuged at 3,000 r.p.m. at room temperature for 15 min. A portion of the surnatant (1 ml) was added to 2 ml of tetrahydrofuran. The resulting mixture was centrifuged at 3,000 r.p.m. for 15 min, the surnatant was collected and analysed by a spectrophotofluorimetric procedure as specified below. In preliminary experiments, we observed that a second treatment of the pellet by the same extraction procedure gave iso-BOSiNc recoveries which were always lower than 5% of the originally extracted amount.

The serum was isolated from blood by centrifugation at 3,000 r.p.m. for 15 min. Fifty microlitres of the serum was added to 700 µl of 2% aqueous SDS and 1.5 ml of tetrahydrofuran, and the mixture was centrifuged at 3,000 r.p.m. for 15 min after which the surnatant was collected and analysed. The fluorescence spectrum of the solutions was recorded at wavelengths above 730 nm ($\lambda_{exc} = 690$ nm). The fluorescence intensity was then converted into iso-BOSiNc concentration (µg of dye per g of tissue or ng of dye per ml of serum) by interpolation with a calibration plot.

*Chromatographic studies*

Chromatographic analyses of sera taken at 2 h after administration of iso-BOSiNc were obtained from mice injected with two doses of photosensitiser (0.5 and 2.0 mg $kg^{-1}$). The serum was taken 2 h after administration of iso-BOSiNc.

**Figure 1** Typical absorption spectrum of Si-(IV)-naphthalocyanine in an aquoseus dispersion (pH 7.4) of small unilamellar liposomes of dipalmitoyl-phosphatidylcholine.

Serum samples were chromatographed on a column (1.7 × 140 cm) of Sephacryl S-300, which had been equilibrated with 0.01 M phosphate buffer at pH 7.4, containing 0.15 M NaCl. The column was eluted at a flow rate of 31.2 ml $h^{-1}$ and 2.6 ml fractions were collected. The fraction collector was connected to a 2238 LKB UV-cord and the protein content was continuously recorded by monitoring the absorbence of the eluate at 280 nm. The collected fractions were also assayed for 780 nm fluorescence emission exciting at 690 nm.

*Discontinuous density gradient ultracentrifugation of sera*

Ultracentrifuge analysis of the distribution of iso-BOSiNc among the various lipoprotein classes were obtained from serum of healthy rabbits injected with 0.5 and 1.0 mg $kg^{-1}$ iso-BOSiNc. The distribution of dyes in rabbit serum is essentially identical to that observed in mouse serum (Jori, 1985). The serum (15 ml) was taken at 2 h after administration.

Ultracentrifugation studies were performed at 39,000 r.p.m. with a Kontron apparatus. The density gradient was obtained by addition of aqueous KBr to the serum. Four density regions were obtained corresponding to four classes of proteins: very low density (VLDL, $d < 1.006$), low density (LDL, $1.006 < d < 1.063$), high density (HDL, $1.063 < d < 1.21$) lipoproteins and a fraction at $d > 1.21$ corresponding to the other serum proteins (bottom).

The purity of the fractions was assayed by agarose gel-electrophoresis. The content of proteins, triglycerides, phospholipids and cholesterol was measured in order to calculate the total lipoprotein mass (holoprotein) (Barel *et al.*, 1986).

The amount of iso-BOSiNc bound with each protein fraction was determined by spectrophotofluorimetric analysis after extraction of the photosensitiser.

*Photodynamic therapy*

For experimental PDT studies we have used tumour-bearing mice injected with 0.5 mg $kg^{-1}$ lipsome-bound iso-BOSiNc. Phototreatments were performed at 24 h after administration of the drug. Light from a 250 W halogen lamp (Teclas, Lugano, Switzerland) was focused into a bundle of optical fibres having a total diameter of 0.6 cm. The fibre tip was placed at 1 cm from the surface of the tumour. The lamp was equipped with a set of optical cut-on and cut-off filters which eliminated all visible and infrared radiation outside the 700–800 nm interval. The development of tumour necrotic area was measured as a function of time after the end of PDT. In general the tumour was irradiated at a dose-rate of 180 mW $cm^{-2}$ (measured at the end of the fibre bundle) and a total light dose of 450 J $cm^{-2}$. The extent of tumour necrotic area was also measured as a function of the irradiation dose-rate in order to define the optimal phototherapeutic parameters. In this case the tumour was irradiated at a total light dose of 450 J $cm^{-2}$ and the necrotic area was analysed at 24 h after the end of PDT. The procedure for measuring the extent of tumour necrosis involved the fixation of the tumour in 10% formalin, followed by sectioning the tumour at 2 mm intervals. The width and depth of the necrotic area were measured for each tissue slice. The maximum values of width and depth were recorded for each tumour and their product was used as a quantitative evaluation of the tumour response (Reddi *et al.*, 1990). Each point reported in Figures 4 and 5 represents the mean (± s.d.) from three animals.

### Results

*Pharmacokinetic studies*

The recovery of iso-BOSiNc from tumour, serum and selected normal tissues at different times after injection of 2 mg $kg^{-1}$ is reported in Table I. The data represent the average of recoveries from at least three different mice, the largest deviation from the reported values being 15%. The clearance of the drug from the serum follows biphasic kinetics; about 90% of photosensitiser is eliminated during the initial 24 h. Significant concentrations of photosensitiser, of the order of μg per g of tissue, are accumulated and retained by the tumour. On the other hand, the peritumoral tissue (i.e. muscle) accumulates significantly smaller amounts of iso-BOSiNc and the ratio of iso-BOSiNc concentration in tumour and muscle ranges between 10 and 20. Unusually large amounts of iso-BOSiNc are recovered in the liver and spleen and they are present even at 1 week after administration. Pharmacokinetic studies with normal mice show that large concentrations of iso-BOSiNc are recovered from liver and spleen also at 4 weeks after injection of 2 mg $kg^{-1}$ (Table III). Moreover, the iso-BOSiNc concentration in the skin shows a tendency to increase at long times after injection. This fact may be related with the prolonged retention of liposome-associated drugs by serum high-density lipoproteins (Jori, 1987), which could release iso-BOSiNc to cutaneous districts.

At a lower injected dose of photosensitiser (0.5 mg $kg^{-1}$) we find again a high ratio of iso-BOSiNc concentration in the tumour and muscle (Table II). However, the amounts of drug taken up by the liver and spleen is substantially reduced and a gradual elimination of iso-BOSiNc from these tissues can be observed. Similar pharmacokinetic data are obtained for normal mice injected with 0.5 mg $kg^{-1}$: as shown in Table III the photosensitiser is slowly eliminated from the liver while there are similar concentrations of iso-BOSiNc in the spleen at times between 1 and 4 weeks after injection.

**Table I** Recoveries of iso-BOSiNc from tumour-bearing BALB/c mice injected with 2 mg $kg^{-1}$ of dye

| | Time lapse after injection | | | | |
|---|---|---|---|---|---|
| | *3 h* | *12 h* | *24 h* | *48 h* | *1 week* |
| Serum | 8060.01 | 1196.51 | 639.39 | 159.32 | 5.21 |
| Tumour | 0.97 | 1.74 | 1.20 | 1.25 | 0.32 |
| Muscle | 0.12 | 0.10 | 0.19 | 0.04 | 0.03 |
| Liver | 6.99 | 5.99 | 12.03 | 13.96 | 7.39 |
| Skin | 0.16 | 0.12 | 0.18 | 0.20 | 0.30 |
| Brain | 0.08 | 0.02 | 0.01 | 0.00 | 0.00 |
| Lung | 1.04 | 0.35 | 0.28 | 0.28 | 0.28 |
| Spleen | 5.79 | 5.38 | 1.82 | 3.47 | 1.38 |
| Kidney | 1.19 | 0.48 | 0.65 | 0.63 | 0.51 |

Data expressed as μg of iso-BOSiNc per g of tissue or ng of iso-BOSiNc per ml of serum (average of three mice).

**Table II** Recoveries of iso-BOSiNc from tumour-bearing BALB/c mice injected with 0.5 mg $kg^{-1}$ of dye

| | Time lapse after injection | | | | |
|---|---|---|---|---|---|
| | *3 h* | *12 h* | *24 h* | *48 h* | *1 week* |
| Serum | 1731.29 | 354.91 | 196.21 | 23.08 | 0.00 |
| Tumour | 0.28 | 0.30 | 0.34 | 0.11 | 0.09 |
| Muscle | 0.11 | 0.04 | 0.02 | 0.00 | 0.00 |
| Liver | 4.09 | 4.81 | 3.61 | 5.94 | 1.78 |
| Skin | 0.01 | 0.02 | 0.02 | 0.01 | 0.03 |
| Spleen | 0.76 | 1.40 | 1.40 | 0.94 | 0.57 |

Data expressed as μg of iso-BOSiNc per g of tissue or ng of iso-BOSiNc per ml of serum (average of three mice).

**Table III** Recoveries of iso-BOSiNc from healthy mice injected with 2.0 or 0.5 mg $kg^{-1}$ of dye

| *Tissue* | *Injected dose (mg $kg^{-1}$)* | Time lapse after injection | | |
|---|---|---|---|---|
| | | *1 week* | *2 weeks* | *4 weeks* |
| Serum | 2 | n.d. | 13.54 | 0.00 |
| Skin | 2 | n.d. | 0.21 | 0.12 |
| Liver | 2 | 9.55 | 10.55 | 8.42 |
| Spleen | 2 | 7.22 | 9.87 | 8.94 |
| Serum | 0.5 | 0.00 | 0.00 | 0.00 |
| Skin | 0.5 | 0.04 | 0.04 | 0.02 |
| Liver | 0.5 | 5.27 | 4.87 | 2.67 |
| Spleen | 0.5 | 2.93 | 3.09 | 3.65 |

Data expressed as μg of iso-BOSiNc per g of tissue or ng of iso-BOSiNc per ml of serum (average of three mice). n.d.: not determined.

**Table IV** Distribution of iso-BOSiNc among the various lipoprotein classes of the serum of healthy rabbits injected with 0.5 or 1.0 mg $kg^{-1}$ of dye

| *Protein* | *Injected dose (mg $kg^{-1}$)* | *% iso-BOSiNc* | *iso-BOSiNc/ apoprotein* | *iso-BOSiNc/ holoprotein* |
|---|---|---|---|---|
| VLDL | 0.5 | 2.63 | 239.61 | 71.50 |
| | 1.0 | 2.39 | 550.65 | 217.08 |
| LDL | 0.5 | 57.79 | 5079.44 | 1231.54 |
| | 1.0 | 48.01 | 5770.06 | 1094.50 |
| HDL | 0.5 | 35.68 | 485.35 | 396.40 |
| | 1.0 | 48.05 | 4856.78 | 2401.10 |
| Bottom | 0.5 | 3.89 | 3.96 | 3.92 |
| | 1.0 | 1.55 | 3.18 | 3.13 |

The serum was taken at 2 h after injection. Data expressed as % of total recovery of iso-BOSiNc or ng of iso-BOSiNc per mg of protein.

*Chromatographic and ultracentrifuge studies*

The chromatograms of the serum obtained from mice injected with 2.0 mg $kg^{-1}$ and 0.5 mg $kg^{-1}$ iso-BOSiNc are shown in Figures 2 and 3, respectively. For serum proteins the absorbence peak 'A' represents an unidentified high

**Figure 2** Chromatogram of the serum of BALB/c mice 2 h after the administration of liposomal iso-BOSiNc at a dose of 2 mg $kg^{-1}$. The chromatogram shows the absorbence at 280 nm (●---●) and the fluorescence emission at 780 nm (▲---▲).

**Figure 3** Chromatogram of the serum of BALB/c mice 2 h after the administration of liposomal iso-BOSiNc at a dose of 0.5 mg $kg^{-1}$. The chromatogram shows the absorbence at 280 nm (●---●) and the fluorescence emission at 780 nm (▲---▲).

molecular weight class of proteins, the peak 'B' represents the lipoproteins and the peak 'C' represents mainly albumin and globulins (Barel *et al.*, 1986). Clearly, the dye is distributed among all serum proteins, although the fraction associated with peak 'A' is substantially reduced upon injection of 0.5 mg $kg^{-1}$. In all cases, the absorption and fluorescence properties of iso-BOSiNc in the different fractions were typical of the monomeric dye.

The results of ultracentrifuge analysis of the distribution of iso-BOSiNc among the various lipoprotein classes are shown in Table IV.

*Phototherapy studies*

Our PDT protocol causes the appearance of tumour necrosis within about 12 h from the end of phototreatment. On the other hand, no appreciable photodamage (e.g. oedema, erythema or ulceration) is observed in the skin overlying or adjacent to the tumour.

Figure 4 shows the extent of tumour necrotic area as a function of time after the end of PDT. The necrotic area reaches a maximum level (total necrosis) at about 24 h after the end of PDT. In Figure 5 we show the extent of tumour necrosis as a function of the irradiation dose rate. Apparently, tumour necrosis increases with increasing dose-rate; in particular, the necrotic area and dose rate (in the range between 100 mW $cm^{-2}$ and 180 mW $cm^{-2}$) seem to be linearly correlated.

**Figure 4** Development of tumour necrosis as a function of time after PDT of MS-2 fibrosarcoma. PDT was performed at 24 h after injection of 0.5 mg $kg^{-1}$ iso-BOSiNc. Irradiation dose-rate was 180 mW $cm^{-2}$, total light dose was 450 J $cm^{-2}$.

**Figure 5** Effect of irradiation dose-rate on the extent of the necrotic area obtained by PDT of MS-2 fibrosarcoma at 24 h after injection of 0.5 mg $kg^{-1}$ iso-BOSiNc. Total light dose was 450 J $cm^{-2}$. The necrosis was measured at 24 h after the end of PDT.

**Discussion**

Our pharmacokinetic data show that significant concentrations of iso-BOSiNc are accumulated and slowly eliminated by our tumour model. On the other hand, the muscle accumulates very small amounts of iso-BOSiNc, so that quite large tumour/muscle ratios of photosensitiser concentration are found. This fact should guarantee a safe phototherapeutic index, at least in our tumour model, namely a minimal risk of photodamage to tumour-adjacent tissues. At the same time, low iso-BOSiNc amounts are recovered from the skin, which should minimise the risk of cutaneous photosensitivity. No significant amounts of iso-BOSiNc are recovered from brain, in agreement with the reported inability of polycyclic aromatic compounds to cross the blood–brain barrier (Zhou, 1989). As a consequence, iso-BOSiNc should not induce toxic effects at the level of the central nervous system. The very small amounts of drug accumulated by kidneys suggest that iso-BOSiNc (as other photosensitisers) is mostly eliminated from the organism via the bile–gut pathway (Reddi *et al.*, 1987). Actually, large amounts of the drug are present in the liver and in the spleen even at one week after administration of 2 mg $kg^{-1}$ iso-BOSiNc. The prolonged retention of this photosensitiser by the main components of the reticuloendothelial system is further confirmed by our pharmacokinetic studies with normal mice. On the other hand, we observe a faster kinetics of iso-BOSiNc elimination by the liver and the spleen after injection of a lower dose (0.5 mg $kg^{-1}$).

This fact is possibly connected with the effect of injected dose on the distribution of the photosensitiser among the serum proteins: the chromatographic and ultracentrifuge analyses of sera obtained from mice and rabbits injected with iso-BOSiNc doses in the range 0.5–2.0 mg $kg^{-1}$ indicate that the drug is mainly transported by the LDL at the lower injected doses, while iso-BOSiNc is largely associated with other proteins at the higher doses.

As known, HDL are responsible for transport and clearance of cholesterol from the components of the reticuloendothelial system. Therefore, maximum selectivity of tumour targeting by iso-BOSiNc is obtained by injection of 0.5 mg $kg^{-1}$ drug. Actually, the ultracentrifuge analysis of the distribution of iso-BOSiNc among the different classes of lipoproteins and other serum proteins shows that only a small fraction of photosensitiser (3.89%) is recovered in the bottom fraction in agreement with the results of chromatographic analysis of the serum after injection of 0.5 mg $kg^{-1}$ iso-BOSiNc. The largest amount of iso-BOSiNc is associated with LDL (57.8%) and to a lesser extent (35.7%) with HDL. This distribution is very significant since LDL represent the protein class mainly responsible for the release of some hydrophobic photosensitising agents to tumour tissues through a mechanism of receptor-mediated endocytosis (Jori, 1987).

PDT experiments show that the irradiation of tumour at 24 h after injection of 0.5 mg $kg^{-1}$ iso-BOSiNc induces an extensive necrosis which reaches a maximum level (total necrosis) at about 24 h after the end of the phototreatment, when dose-rates as large as 180 mW $cm^{-2}$ are used. In this connection, the phototherapeutic efficiency of iso-BOSiNc is comparable with that observed upon injection of the same tumour model with 0.2 mg $kg^{-1}$ Zinc(II)-phthalocyanine or 10 mg $kg^{-1}$ Hp (Cozzani *et al.*, 1984; Reddi *et al.*, 1990). The observed increase of the photodamaged tumour area upon increasing the dose-rate probably does not reflect a significant contribution from thermal damage. Actually irradiation of tumour tissues in uninjected mice at 180 mW $cm^{-2}$ caused no detectable damage. Under these experimental conditions, the increase of tissue temperature above the basal level (29–30°C) for the anaesthetised mice was not greater than 5°C, which is lower than that usually considered to originate hyperthermal effects (Evensen & Moan, 1988). Henderson and Mayhew (1990) observed a good response of subcutaneously implanted RIF (radiation induced fibrosarcoma) in iso-BOSiNc treated mice to light doses (135 J $cm^{-2}$, 75–100 mW $cm^{-2}$) lower than those used in the present study. This can be explained by several differences existing between the two experimental protocols: the tumour type and location (intramuscolar *vs* subcutaneous) often cause different levels of photosensitivity; moreover, the liposomal carriers of iso-BOSiNc were different, which may result in different modalities of photosensitiser transport in the serum and different distribution of the photosensitiser among the tissular compartments (Ginevra *et al.*, 1990). Actually, Henderson and Mayhew (1990) found a large extent of PDT-induced vascular damage in their tumour animal model, while ultrastructural studies on tissue specimens taken from the irradiated MS-2 fibrosarcoma demonstrate a highly preferential direct damage of malignant cells (Jori & Cuomo, unpublished observations).

This work was supported in part by NIH grant CA 46281 and by the Center for Photochemical Sciences at Bowling Green State University, and in part by CNR (Italy), special project Oncologia, grant no. 88.0710.44.

### References

BAREL, A., JORI, G., PERIN, A., ROMANDINI, P., PAGNAN, A. & BIFFANTI, S. (1986). Role of high-, low- and very low-density lipoproteins in the transport and tumor-delivery of hematoporphyrin in vivo. *Cancer Lett.*, **32**, 145.

COZZANI, I., JORI, G., REDDI, E., TOMIO, L. & SICURO, T. (1984). Interaction of free and liposome-bound porphyrins with normal and malignant cells: biochemical and photosensitization studies in vitro and in vivo. In *Tumour Phototherapy*, Andreoni, A. & Cubeddu, R. (eds) p. 157. Plenum Press: New York.

DOUGHERTY, T.J. (1987). Photosensitizers: therapy and detection of malignant tumors. *Photochem. Photobiol.*, **45**, 879.

EVENSEN, J.F. & MOAN, J. (1988). Photodynamic therapy of C3H tumours in mice: effect of drug/light dose fractionation and misonidazole. *Laser Med. Sci.*, **3**, 1.

FIREY, P.A. & RODGERS, M.A.J. (1987). Photoproperties of silicon naphthalocyanine, a potential photosensitizer for photodynamic therapy. *Photochem. Photobiol.*, **45**, 535.

FORD, W.E., FIREY, P.A., SOUNIK, J.R., RIHTER, B., KENNEY, M.E. & RODGERS, M.A.J. (1988). Photoproperties of naphthalocyanines. *Proc. SPIE*, **997**, 105.

GINEVRA, F., BIFFANTI, S., BIOLO, R., REDDI, E. & JORI, G. (1990). Delivery of the tumour photosensitizer zinc (II) – phthalocyanine to serum proteins by different liposomes: studies in vitro and in vivo. *Cancer Lett.*, **49**, 59.

HENDERSON, B.W. & MAYHEW, E. (1990). Experience with the liposomal delivery of the photosensitizer iso-BOSiNc. *Proc. SPIE*, **1203**, 12.

JORI, G. (1985). Pharmacokinetic studies with hematoporphyrin in tumour-bearing mice. In *Photodynamic Therapy of Tumours and Other Diseases*, Jori, G. & Perria, C. (eds) p. 159. Libreria Progetto: Padova.

JORI, G. (1987). Photodynamic therapy of solid tumors. *Radiat. Phys. Chem.*, **30**, 375.

JORI, G., TOMIO, L., REDDI, E., ZORAT, P.L. & CALZAVARA, F. (1983). Preferential delivery of liposome-incorporated porphyrins to neoplastic cells in tumour-bearing rats. *Br. J. Cancer*, **48**, 307.

MOAN, J. (1986). Porphyrin-sensitized photodynamic inactivation of cells. *Laser Med. Sci.*, **1**, 5.

REDDI, E., LO CASTRO, G., BIOLO, R., MENEGALDO, E. & JORI, G. (1987). Pharmacokinetic studies with Zn(II)-phthalocyanine in tumor-bearing mice. *Br. J. Cancer*, **56**, 597.

REDDI, E., ZHOU, C., BIOLO, R., MENEGALDO, E. & JORI, G. (1990). Liposome- or LDL-administered Zn(II)-phthalocyanine as a photodynamic agent for tumours. I. Pharmacokinetic properties and phototherapeutic efficiency. *Br. J. Cancer*, **61**, 407.

TOMIO, L., CALZAVARA, F., ZORAT, P.L. & CORTI, L. (1984). Photoradiation therapy of cutaneous and subcutaneous malignant tumours using hematoporphyrin. In *Prophyrin Localization and Treatment of Tumours*, Doiron, D. & Gomer, C.J. (eds) p. 829. Alan R. Liss: New York.

WHEELER, B.L., NAGASUBRAMANIAN, G., BARD, A.J., SCHECHTMAN, L.A., DININNY, D.R. & KENNEY, M.E. (1984). A silicon phthalocyanine and a silicon naphthalocyanine: synthesis, electrochemistry and electrogenerated chemiluminescence. *J. Am. Chem. Soc.*, **106**, 7404.

WILSON, B.C. & JEEVES, W.P. (1987). Photodynamic therapy of cancer. In *Photomedicine, vol. 2*, Ben-Hur, E. & Rosenthal, I. (eds) p. 127. CRC Press: Boca Raton, FL.

ZHOU, C. (1989). Mechanism of tumour necrosis induced by photodynamic therapy. *J. Photochem. Photobiol. B (Biol.)*, **3**, 299.